# Complete chloroplast genome sequence and phylogenetic analysis of *Syzygium malaccense*

**DOI:** 10.1080/23802359.2020.1829132

**Published:** 2020-10-09

**Authors:** Liang Tao, Zhuo-Gong Shi, Qing-Yi Long

**Affiliations:** a Southwest Forestry University, Kunming, China; b Yunnan Institute of Tropical Crops, Xishuangbanna, China

**Keywords:** *Syzygium malaccense*, *Syzygium malaccense*, chloroplast genome, phylogenetic analysis

## Abstract

*Syzygium malaccense* is native to Malaysia. It is sometimes called the malay apple, malay rose-apple, mountain rose-apple, mountain apple, water apple, or French cashew. The tree is very popular in many tropical and subtropical regions for its fruit and traditional medicine. The first complete chloroplast genome of *Syzygium malaccense* has been reported in this study. The complete chloroplast genome of *Syzygium malaccense* is 158,954 bp, composed of four regions: a large single-copy region with a size of 87,991 bp, a small single copy region with a size of 18,793 bp, and two inverted repeat regions with a size of 26,085 bp. The GC content is 36.97%. A total of 132 genes were annotated, including 84 encoding proteins, eight encoding rRNA genes, 37 encoding tRNA genes, and three encoding pseudo genes. Phylogenetic analysis showed that *Syzygium aromaticum*, *Syzygium cumini,* and *Syzygium forrestii* are closely related to *Syzygium malaccense*.

*Syzygium malaccense* is native to Malaysia and has been admired for the tree’s beauty. It is sometimes called the malay apple, malay rose-apple, mountain rose-apple, mountain apple, water apple, or French cashew (Sankat et al. 2000; Oliveira et al. 2011). The fruits of *Syzygium malaccense* are pyriform with external colors of yellow, pink, or purple, and the soft flesh of the ripe fruit has a pleasant sweet flavor with a characteristic rose aroma (Pino et al. 2004). The edible rate of *Syzygium malaccense* ripe fruit is more than 80%, and its fruit have high nutritional value. The fruits can be used as raw materials for fruit paste, candied fruit, or jam. Fermented fruit juice can also be brewed into a high-grade drink. In addition to their fruit, various parts of the plant have also been applied in traditional medicine (Arumugam et al. 2014). Different plant parts of *Syzygium malaccense* have been widely used to treat diabetes traditionally in Brazil (Whistler and Elevitch 2006), its bark extract has been shown to effectively serve as a hypoglycemic agent that improved the fasting blood-sugar level and the liver-glycogen depletion and reduced diabetes-induced hyperlipidemia in diabetic rats (Bairy et al. 2005), the plant extract has been shown to be a good inhibitor of aldose reductase, lens enzyme that is involved in the development of diabetes-induced cataractogenesis (Guzman and Guerrero 2005), and the myricetin derivatives isolated from leaf extract of *Syzygium malaccense* exhibited 'insulin-like' effects by enhancing accumulation of lipid, glucose uptake and adiponectin secretion by activating insulin signaling pathway similar to insulin (Arumugam et al. 2016). For these reasons, the plant is very popular in many tropical and subtropical regions. There have been numerous reports on *Syzygium malaccense* in recent years, but the chloroplast genome has not yet been reported yet. In this study, the first complete chloroplast genome of *Syzygium malaccense* is reported.

Tender leaves of *Syzygium malaccense* were collected from the Tropical Fruit Garden of the Yunnan Institute of Tropical Crops (22.01566069°N 100.78952540°E) and the specimen was deposited in the herbarium of Yunnan Institute of Tropical Crops (Xishuangbanna, China) with the voucher number of YITC/SFU/TF-2020-0196. High-quality genomic DNA was isolated using a DNeasy Plant Mini Kit (Qiagen, Venlo, The Netherlands). DNA library were prepared with the insert sizes of 350 bp and paired-end (PE)sequencing was conducted on the Illumina Hi-Seq 2500 Platform (Illumina, San Diego, CA, USA) to assemble the chloroplast genome. Low-quality reads and adapters were removed by FastQC (Andrews 2015) and the chloroplast genome was assembled by NOVOPlasty(v.2.7.2) (Dierckxsens et al. 2016). The chloroplast genome was annotated by Geneious8.1.7 (Kearse et al. 2012) and corrected by DOGMA (Wyman et al. 2004), the complete chloroplast genome of *Syzygium aromaticum* (NC_047249) was used as the reference genome for assembly and annotation, the gaps and boundaries of IRs were confirmed by PCR assays. The results of the chloroplast genome assembly and annotation were uploaded to GenBank (http://www.ncbi.nlm.nih.gov/) with the accession number MT830744.

The complete chloroplast genome of *Syzygium malaccense* is 1,58,954 bp long. Like those of other plants, the genome is also composed of four regions: a large single-copy region (LSC) with a length of 87,991 bp and a small single copy region (SSC) with a length of 18,793 bp separated by two inverted repeat regions (IRs) with a length of 26,085 bp. The whole chloroplast genome is composed of 31.11% A, 31.91% T, 18.12% G, and 18.85% C nucleotides, and the GC content is 36.97%. A total of 132 genes were annotated, including 84 encoding proteins, 8 encoding rRNA genes, 37 encoding tRNA genes, and 3 encoding pseudo genes.

To confirm the phylogenetic relationship of the *Syzygium malaccense* with other species of plants, complete chloroplast genome sequences of *Syzygium malaccense* and 25 other species of the Myrtaceae family were aligned using MAFFT (Katoh and Standley 2013), and *Salvertia convallariodora* which belongs to the Vochysiaceae family was used as the outgroup. A maximum likelihood tree was constructed using MEGA7.0 (Kumar et al. 2016), the GTR + G+Inucleotide substitution model was selected for the ML tree construction, and the bootstraps value was 1000. The ML phylogenetic tree (Figure 1) showed that *Syzygium aromaticum*, *Syzygium cumini,* and *Syzygium forrestii* is closely related to *Syzygium malaccense*. This study provides abundant genomic data for the research and development of *Syzygium malaccense*.

**Figure 1.**
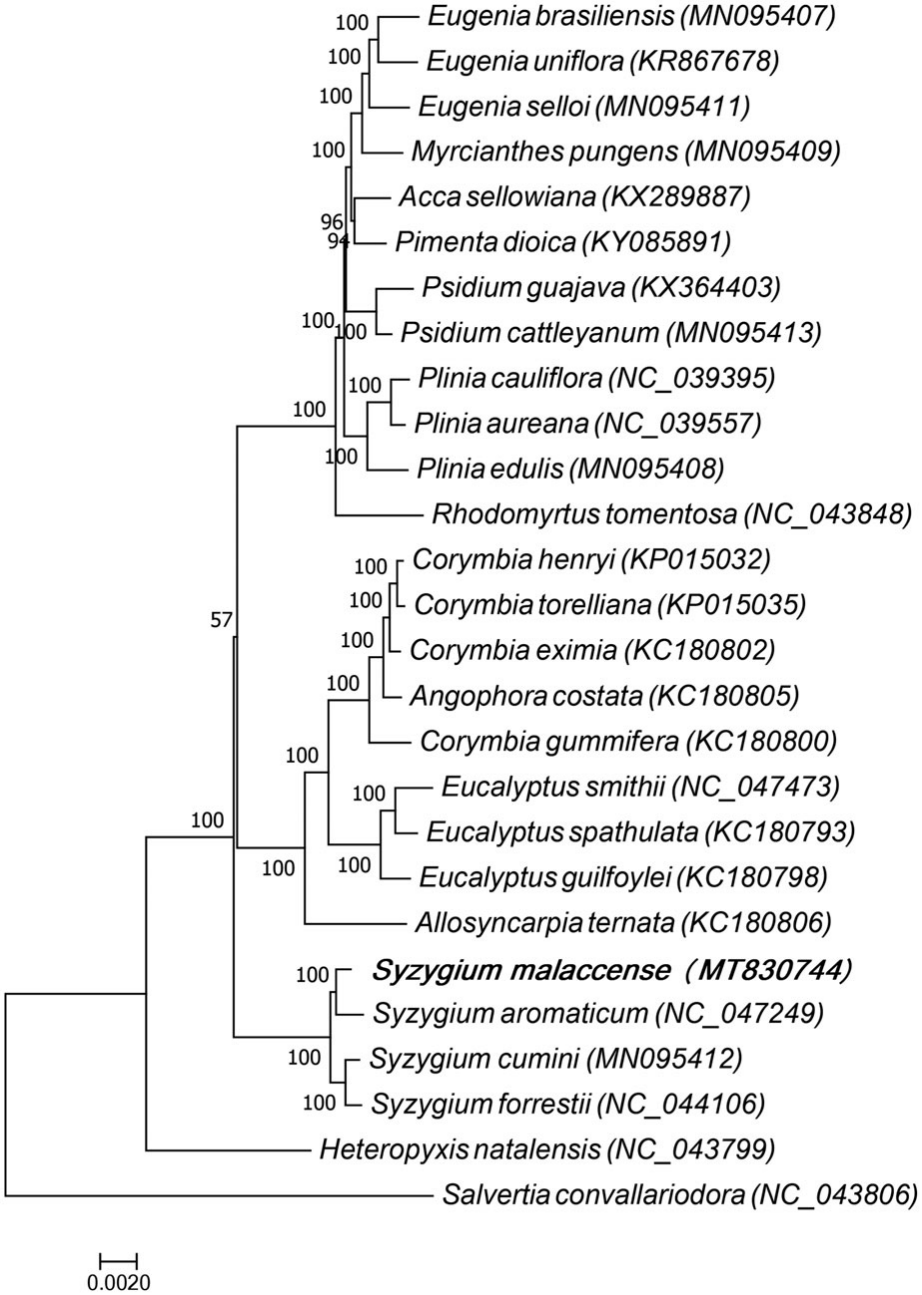
Phylogenetic tree of *Syzygium malaccense* and 25 other species of the Myrtaceae family, and *Salvertia convallariodora* which belongs to the Vochysiaceae family was used as the outgroup. The 27 species for phylogenetic tree construction are: *Eugenia brasiliensis* (MN095407), *Eugenia uniflora* (KR867678), *Eugenia selloi* (MN095411), *Myrcianthes pungens* (MN095409), *Acca sellowiana* (KX289887)*, Pimenta dioica* (KY085891), *Psidium guajava* (KX364403), *Psidium cattleyanum* (MN095413), *Plinia cauliflora* (NC_039395), *Plinia aureana* (NC_039557), *Plinia edulis* (MN095408), *Rhodomyrtus tomentosa* (NC_043848), *Corymbia henryi* (KP015032), *Corymbia torelliana* (KP015035), *Corymbia eximia* (KC180802), *Angophora costata* (KC180805), *Corymbia gummifera* (KC180800), *Eucalyptus smithii* (NC_047473), *Eucalyptus spathulata* (KC180793), *Eucalyptus guilfoylei* (KC180798), *Allosyncarpia ternata* (KC180806), *Syzygium malaccense* (MT830744), *Syzygium aromaticum* (NC_047249), *Syzygium cumini* (MN095412), *Syzygium forrestii* (NC_044106), *Heteropyxis natalensis* (NC_043799), and *Salvertia convallariodora* (NC_043806).

## Data Availability

The data that support the findings of this study are openly available in GenBank at https://www.ncbi.nlm.nih.gov/Genbank/, reference number MT830744. Raw sequencing reads used in this study have been deposited in SRA with the accession PRJNA658814.
